# Supplementary material for: Ten-year follow-up of auditory brainstem implants: From intra-operative electrical auditory brainstem responses to perceptual results
Source: PLoS One. 2023 Mar 2;18(3):e0282261. doi: 10.1371/journal.pone.0282261 (PMC9980821; doi:10.1371/journal.pone.0282261)
Supplement: S1 File — (DOCX) [file pone.0282261.s002.docx]

Multinomial logistic regression models.

. mlogit perceptiveresultsfollowup10y elactivation i.children1adults2 if perceptiveresultsfollowup10y > 0, baselev b(1)

Iteration 0: log likelihood = -15.909027

Iteration 1: log likelihood = -11.439737

Iteration 2: log likelihood = -10.645582

Iteration 3: log likelihood = -10.505157

Iteration 4: log likelihood = -10.474014

Iteration 5: log likelihood = -10.467787

Iteration 6: log likelihood = -10.466476

Iteration 7: log likelihood = -10.466174

Iteration 8: log likelihood = -10.466101

Iteration 9: log likelihood = -10.466087

Iteration 10: log likelihood = -10.466084

Iteration 11: log likelihood = -10.466084

Multinomial logistic regression Number of obs = 15

LR chi2(4) = 10.89

Prob > chi2 = 0.0279

Log likelihood = -10.466084 Pseudo R2 = 0.3421

----------------------------------------------------------------------------------

perceptivere~10y | Coefficient Std. err. z P>|z| [95% conf. interval]

-----------------+----------------------------------------------------------------

1 | (base outcome)

-----------------+----------------------------------------------------------------

2 |

elactivation | .7364072 .4072515 1.81 0.071 -.0617911 1.534605

|

children1adults2 |

1 | 0 (base)

2 | -20.8944 4562.932 -0.00 0.996 -8964.078 8922.289

|

_cons | 8.311434 4562.935 0.00 0.999 -8934.876 8951.499

-----------------+----------------------------------------------------------------

3 |

elactivation | .4979229 .3407789 1.46 0.144 -.1699916 1.165837

|

children1adults2 |

1 | 0 (base)

2 | -20.15516 4562.932 -0.00 0.996 -8963.338 8923.028

|

_cons | 12.32758 4562.933 0.00 0.998 -8930.858 8955.513

----------------------------------------------------------------------------------

. mlogit perceptiveresultsfollowup10y elactivation i.children1adults2 if perceptiveresultsfollowup10y > 0 & var10=="", baselev b(1)

Iteration 0: log likelihood = -13.752771

Iteration 1: log likelihood = -9.8840181

Iteration 2: log likelihood = -9.4182355

Iteration 3: log likelihood = -9.3326512

Iteration 4: log likelihood = -9.3145403

Iteration 5: log likelihood = -9.3107801

Iteration 6: log likelihood = -9.3101336

Iteration 7: log likelihood = -9.3099847

Iteration 8: log likelihood = -9.3099485

Iteration 9: log likelihood = -9.3099413

Iteration 10: log likelihood = -9.3099401

Iteration 11: log likelihood = -9.3099398

Multinomial logistic regression Number of obs = 13

LR chi2(4) = 8.89

Prob > chi2 = 0.0640

Log likelihood = -9.3099398 Pseudo R2 = 0.3230

----------------------------------------------------------------------------------

perceptivere~10y | Coefficient Std. err. z P>|z| [95% conf. interval]

-----------------+----------------------------------------------------------------

1 | (base outcome)

-----------------+----------------------------------------------------------------

2 |

elactivation | .5794921 .382872 1.51 0.130 -.1709233 1.329907

|

children1adults2 |

1 | 0 (base)

2 | -19.10077 4473.215 -0.00 0.997 -8786.442 8748.24

|

_cons | 9.40249 4473.218 0.00 0.998 -8757.943 8776.748

-----------------+----------------------------------------------------------------

3 |

elactivation | .5569244 .3524029 1.58 0.114 -.1337725 1.247621

|

children1adults2 |

1 | 0 (base)

2 | -19.69835 4473.215 -0.00 0.996 -8787.039 8747.643

|

_cons | 10.81208 4473.217 0.00 0.998 -8756.532 8778.156

----------------------------------------------------------------------------------
